# Whole-genome amplification as a tool to improve bacterial detection by PCR in microbiological samples after endodontic treatment

**DOI:** 10.3389/froh.2025.1520945

**Published:** 2025-02-03

**Authors:** Gabriela Ceccon Chianca, Caroline Corrêa Fendeler, Sérgio Pinto Chaves Junior, Gabriella Lorena Dias Pereira, Helvécio Cardoso Corrêa Póvoa, Lívia Azeredo Alves Antunes, Leonardo Santos Antunes, Natalia Lopes Pontes Póvoa Iorio

**Affiliations:** ^1^ Experimental and Applied Microbiology Laboratory, Nova Friburgo Institute of Health, Universidade Federal Fluminense (UFF), Nova Friburgo, Rio de Janeiro, Brazil; ^2^ Graduate Program in Pathology, School of Medicine, UFF, Niterói, Rio de Janeiro, Brazil; ^3^ Graduate Program in Dentistry, Nova Friburgo Institute of Health, UFF, Nova Friburgo, Rio de Janeiro, Brazil; ^4^ Graduate Program in Applied Microbiology and Parasitology, Biomedical Institute, UFF, Niterói, Rio de Janeiro, Brazil; ^5^ Department of Specific Formation, Nova Friburgo Institute of Health, UFF, Nova Friburgo, Rio de Janeiro, Brazil; ^6^ Graduate Program in Dentistry, School of Dentistry, UFF, Niterói, Rio de Janeiro, Brazil

**Keywords:** dental pulp necrosis, microbiology, nucleic acid amplification techniques, polymerase chain reaction, bacteria

## Abstract

**Introduction:**

Microorganisms have an important role in the pathogenesis of endodontic disease. Significant advances have been made to increase the sensitivity of microbial detection, identification and enumeration in endodontic samples. The aim of the present study is to compare culture and whole-genome amplification (WGA) followed by PCR assays in the detection of bacteria before and after chemical mechanical preparation (CMP) of root canals.

**Methods:**

Ten uniradicular teeth with primary endodontic infections were analyzed. Microbiological samples were collected before and after CMP using paper points, which were separated into two groups: (i) culture assay samples were plated onto Brucella agar with 5% defibrinated sheep's blood, menadione and hemin and incubated anaerobically for 14 days at 36°C; (ii) DNA was extracted from molecular assay samples and subject to WGA by isothermal strand displacement with Phi29 DNA polymerase followed by PCR to determine the presence of bacteria.

**Results:**

In both assays, samples before CMP showed the presence of bacteria in all 10 teeth. After CMP, however, bacterial detection differed in the assays performed (*p* = 0.0198). The presence of bacteria was detected in 70% (7 of 10) of the samples by WGA followed by PCR, where only 10% (1 of 10) had demonstrated bacterial growth in the culture method.

**Conclusion:**

The combination of WGA followed by PCR increased the detection of microorganisms from root canal samples after endodontic treatment using NaOCl as a CMP irrigant. So this combination of techniques can represent an important tool to improve the detection of microorganisms in endodontic research.

## 1 Introduction

Apical periodontitis is one of the most common endodontic diseases, resulting from an inflammatory response triggered by host defense reactions primarily due to multiple microorganisms present in the infected root canal system (1). A systematic review revealed that 41% of teeth with root canal treatment had apical periodontitis, and 3.5% of untreated teeth had this disease (2). Patients requiring endodontic treatment often experience a compromised quality of life related to oral health (3, 4).

The primary objectives of endodontic treatment for teeth with infected root canals are to reduce the amount of microorganisms within the root canals and prevent re-infection (5, 6). Instrumentation and irrigation procedures, also called Chemical Mechanical Preparation (CMP), are essential for achieving effective cleansing of the root canal system (7). Inappropriate mechanical debridement is one of the commonly attributable causes of endodontic failure (8).

Microorganisms have an important role in the pathogenesis of endodontic disease (5), as many cultivable and non-cultivable microorganisms are present in the infected root canal environment and contribute to the progression of this disease (9–11). The infection develops as the pulp loses its vitality and apical periodontitis ensues (12, 13).

The microbial diversity involved in endodontic disease (10, 12–16) accounts for the wide range of methods for detecting these microorganisms. Such methods are used in clinical and laboratorial studies, to better comprehend the endodontic microbiota as well as to evaluate the antimicrobial effects of several endodontic treatments that aim to avoid endodontic failure (15, 17–20).

Significant advances, using molecular biology techniques, have been made in the last decades to increase the sensitivity of microbial detection, identification and enumeration in endodontic samples, such as: use of PCR, quantitative real-time PCR, checkerboard DNA–DNA hybridization, sequencing, as well as an association of whole-genome amplification (WGA) with some of these methodologies (10, 15, 16, 21–23). Advanced analytical tools may contribute to addressing current gaps in understanding the mechanisms behind endodontic pathogenesis and response to treatment (5).

WGA is a technique that amplifies the whole genome to generate an abundant amount of DNA with accuracy, preserving the genomic sequence and genotypic information (24). The amplified product of this reaction can be used directly in most methods of DNA analysis (25). WGA has a large variety of applicabilities, such as forensics and paleontology being widely used in molecular diagnosis and medicine especially when there is a small amount of starting material (26).

In endodontics, studies have used WGA to increase the sensitivity of the subsequent species-specific PCR (23) and to find a greater number of bacterial DNA in studies that use checkerboard DNA-DNA hybridization. In the latter, WGA could be used in the step of amplification of the DNA probes as well as in the step of DNA amplification from clinical samples (10, 15).

Although WGA has been used in few endodontic studies, to date there is still a lack of them comparing this technique associated with subsequent PCR to phenotypic culture methods. The association between WGA and PCR could contribute to endodontic research in differentiating low bacterial loads from the absence of bacteria which could, in turn, guide clinicians during endodontic treatment seeking to decrease endodontic failure. Therefore, the aim of the present study is to compare the culture and WGA followed by PCR assays in the detection of bacteria before and after CMP of root canals from 10 teeth with apical periodontitis, using NaOCl as an irrigant. The null hypothesis is that there would be no difference between the bacterial detection between culture and WGA followed by PCR.

## 2 Materials and methods

### 2.1 Participants

This study was approved by the local research ethics committee (protocol number: 707.933), and written informed consent was obtained from all participants. It was also registered in the international database ClinicalTrials.gov (NCT03212729).

Nine patients were selected from April 2015 to June 2015, in the dental clinic of a Brazilian public university. Each patient contributed with one tooth, except one, who contributed with two teeth, totalizing 10 teeth with apical periodontitis. The participants were from both genders with ages ranging between 17 and 65 years old.

The inclusion criteria were teeth with: a single canal with endodontic infection, intact pulp chamber walls, necrotic pulp confirmed by pulp sensitivity tests and clinical and radiographic evidence of asymptomatic apical periodontitis.

Exclusion criteria were teeth with: gross carious lesions, root or crown fracture, previous endodontic treatment, gingival recession and periodontal pockets deeper than 4 mm. Patients who were pregnant, breast feeding, with systemic diseases that could compromise the immune system, and individuals who received antibiotic therapy within the previous 3 months or who were in immunosuppressive treatment were also excluded.

### 2.2 Sample size

The sample size was calculated according to the detection of bacteria by PCR before and after CMP using rotary instrumentation with 2.5% NaOCl from a previous study (27). The statistical program BioEstat 5.3 software (Institute for Sustainable Development Mamiraua, Tefe, AM, Brazil, a freeware available at http://www.mamiraua.org.br) was used and a 5% level of significance was adopted as well as 95% power in the binomial one-tailed *t*-test. Thus, the sample reached a minimum of 9 participants.

### 2.3 Sample collection

The microbiological samples, previously isolated by da Silva et al. (19), were taken in two moments: before CMP (samples A) and after CMP (samples B). They were collected by the same operator who performed all clinical procedures (operator: SPCJ), with eight sterile paper points no. 15: four before CMP (samples A1-A4) and four after CMP (samples B1-B4). Each paper point was left in the canal, previously filled with sterile saline solution, for 30 s, approximately 1 mm short of the radiographic root apex.

After local anesthesia using lidocaine 2% with epinephrine 1:50.000 (Dentsply Sirona, Catanduva, SP, Brazil), supragingival calculus and biofilm were removed from each tooth through scaling and cleaning with pumice. A rubber dam (Madeitex Indústria e Comércio de Artefatos de Latex, São José dos Campos, SP, Brazil) was then applied, and carious tissue was removed using a spherical rotary bur no. 4 (KG Sorensen, São Paulo, SP, Brazil) at high rotation using sterile saline as an irrigant. Subsequently, the operating field and teeth were cleaned in sequence using a 3% hydrogen peroxide solution (Rioquímica Indústria Farmacêutica, São José do Rio Preto, SP, Brazil), followed by a 2% iodine solution (Rioquímica Indústria Farmacêutica), another 3% hydrogen peroxide rinse (Rioquímica Indústria Farmacêutica), a 2.5% NaOCl solution (Rioquímica Indústria Farmacêutica), and finally, a 5% sodium thiosulfate solution (Química Moderna, Barueri, SP, Brazil) to neutralize any residual iodine and NaOCl. The access cavity preparation was completed using a sterile bur under sterile saline irrigation. The cleaning protocol was then repeated for the operating field and teeth as described above and the microbiological samples were collected. To verify the efficacy of this protocol two sterility control samples were taken from the cavosurface angle of the access cavity by scrubbing with sterile paper points no. 15.

Root canal instrumentation was performed during the same appointment in all cases. The crown-down technique was employed using Gates Glidden drills (Dentsply Sirona) and Kerr files (Dentsply Sirona) with an anatomic diameter compatible with the root canal. Irrigation was carried out using 5 ml of 2.5% NaOCl solution between each endodontic file. The working length was determined 1 mm short of the radiographic apex. The smear layer was removed by rinsing the canal with a 17% ethylenediaminetetraacetic acid solution (Biodinâmica Química e Farmacêutica LTDA, Ibiporã, PR, Brazil), which was left in the canal for 5 min, followed by a final irrigation with 15 ml of 2.5% NaOCl solution. The canal was then dried with sterile paper points (Endo Points Tanari, Paraíba do Sul, RJ, Brazil) and flushed with 5 ml of 5% sodium thiosulfate solution to neutralize any residual NaOCl before microbiological samples were collected.

A paste containing pure calcium hydroxide paste (Biodinâmica Química e Farmacêutica LTDA) mixed with paramonoclorofenol (Biodinâmica Química e Farmacêutica LTDA) was inserted into the canal with Lentulo spirals size 35 (Dentsply Sirona) at low rotation, ensuring complete filling of the root canal as temporary medication between sessions. Coronal sealing was then achieved with Coltosol (Coltène, Altstatten, SG, Switzerland), followed by glass ionomer cement Maxxion R (FGM Dental Group, Joinville, SC, Brazil).

In the following session, the canal was filled with the hybrid Tagger technique, with a Mc Spadden condenser (Dentsply Sirona). Coronal sealing was then achieved by glass ionomer cement Maxxion R (FGM Dental Group).

### 2.4 Microbiological procedures

The paper points were separated into two groups: culture and molecular (WGA and PCR) assays. The first (samples A1 and B1) and third (samples A3 and B3) paper points were placed in microtubes containing 0.5 ml of 0.9% sterile saline and destined for culture methods. Whereas the second (samples A2 and B2) and fourth (samples A4 and B4) were placed in microtubes containing 0.5 ml of sterile Tris-EDTA buffer solution pH 8.0 (Sigma-Aldrich Chemical Co, St. Louis, MO, USA) and destined for molecular methods. The samples were immediately transported to the Microbiology Laboratory and processed (culture assay) or frozen at −20°C (molecular assays).

#### 2.4.1 Culture assays

Microtubes containing the paper points for the culture assay were homogenized in a tube shaker (Kasvi Produtos Laboratoriais, Pinhais, PR, Brazil) for 30 s/3,300 rpm and 50 μl were plated, in triplicate, onto Brucella agar (Becton, Dickinson and Company, Sparks, MD, USA) with 5% defibrinated sheep's blood, menadione and hemin (operator: NLPPI). The plates were incubated for 14 days at 36°C in an anaerobiosis jar (Becton, Dickinson and Company), using anaerobic gas generating sachets (AnaeroGen™-Oxoid Ltd, Basingstoke, HA, United Kingdom).

The sterility control paper points were immediately placed into Fluid Thioglycollate Medium (Becton, Dickinson and Company) and incubated for 14 days at 36°C in order to observe the presence of cultivable microorganisms (operator: NLPPI). As an inclusion criteria, the teeth in the control samples had to have no microbial growth in these cultures.

#### 2.4.2 Molecular assays: whole-genome amplification (WGA) and PCR

Deep frozen microtubes containing the paper points for the molecular assays were thawed and dispersed by constant stirring for 30 min (160 rpm) (Benfer Produtos para Laboratórios, São Paulo, SP, Brazil). Paper points were discarded and DNA extraction of the microbiological samples was performed using the QIAamp DNA Mini Kit (Qiagen, Valencia, CA, USA), according to the manufacturer's instructions (operator: CCF).

The extracted DNAs were subject to WGA by isothermal strand displacement with Phi29 DNA polymerase using the Illustra GenomiPhi V2 DNA Amplification kit (GE Healthcare, Piscataway, NJ, USA) according to the manufacturer's instructions to increase the amount of DNA. The DNA was quantified using Qubit 2.0 Fluorometer (Life Technologies, Carlsbad, CA, USA), with the Qubit dsDNA HS Assay kit (Life Technologies). All samples with more than 125 ng/µl (maximum sensibility of Qubit 2.0 Fluorometer - Life Technologies) were submitted to a decimal dilution and subject to another read to calculate their initial concentration (operators: CCF and GLDP).

The presence of bacteria in the microbiological samples was determined by endpoint PCR. Aliquots of 10 ng of the extracted DNAs subject to WGA were used in the PCR protocol for microorganisms from Bacteria domain (5'-CCTACGGGAGGCAGCAG-3'/5'-CCGTCAATTCMTTTRAGT-3') (28) (operator: CCF).

Positive and negative controls consisted of DNA extracted from *Enterococcus faecalis* (ATCC 29212) and *Candida albicans* (ATCC 10231), respectively. PCR amplifications were performed in a DNA thermocycler (Life Technologies), analyzed by 1% agarose gel electrophoresis with GelRed 1X (Biotium - Glowing Products for Science™, Hayward, CA, USA) and visualized on a UV transilluminator (Kasvi Produtos Laboratoriais).

The operators (operators: CCF and GLDP) were blinded during all molecular assays.

### 2.5 Statistical analysis

Data was analyzed using the statistical mobile app Epi Info v5.5.9 (Centers for Disease Control and Prevention, Atlanta, GA, USA). Fisher's exact test was applied to compare the results from the culture and PCR methods in the detection of bacteria. Differences were considered significant when values of *p* < 0.05 were obtained.

## 3 Results

Ten permanent teeth with necrotic pulp from nine participants were included in this study. All paper points from sterility control determined the absence of cultivable microorganisms.

After the WGA assay, the amount of DNA ranged from 2.4 to 422 ng/µl. Initially, three of these samples presented less than 0.5 ng/µl, so their yields were submitted to a new WGA and reached concentrations from 4.5 to 22 ng/µl.

Results of the presence of bacterial growth by culture and detection of bacteria domain by molecular (WGA and PCR) assays in the samples before CMP (samples A) and after CMP (samples B) are shown in Table 1.

**Table 1:** Detection of the presence of bacteria in microbiological samples by culture and WGA followed by PCR assays.

Samples	Before CMP			After CMP		
	Culture	WGA + PCR	*p*-value	Culture	WGA + PCR	*p*-value
1	+	+	1.0	-	+	0.0198
2	+	+		-	+	
3	+	+		-	-	
4	+	+		-	+	
5	+	+		+	+	
6	+	+		-	-	
7	+	+		-	-	
8	+	+		-	+	
9	+	+		-	+	
10	+	+		-	+	

CMP, chemical mechanical preparation; WGA, whole-genome amplification; PCR, polymerase chain reaction; +, presence of bacteria; -, absence of bacteria.

In both assays, the analysis of the samples before CMP (sample A) showed the presence of bacteria in all ten teeth (100%). After CMP (sample B), the presence of bacteria was detected in 7 teeth (70%) by WGA followed by PCR, where only one tooth (10%) had demonstrated bacterial growth in the culture method. The controls used in the molecular assays showed presence and absence of bacteria, for *Enterococcus faecalis* and *Candida albicans*, respectively.

No significant difference was found for the detection of bacteria by the culture and WGA followed by PCR assays in the samples before CMP (*p* = 1). In contrast, in the microbiological samples collected after CMP, a significant difference was found for the detection of bacteria amongst the two assays (*p* = 0.0198), therefore rejecting the null hypothesis.

## 4 Discussion

Several studies in dentistry use microbiological assays as an analytical tool (29–33) to evaluate the success of endodontic treatment (19, 20, 27, 34, 35).

However it is widely recognized that, due to the complex anatomy of the root canal system, microorganism eradication is utopic in most cases (36), and it is not uncommon to find studies with negative cultures in root canals after CMP (18, 27, 37). To improve bacterial detection sensitivity, some studies use other methods to look for bacteria in endodontic canals, such as: cryopulverization followed by qPCR (17); multiple displacement amplification combined with checkerboard DNA-DNA hybridization (15); WGA before PCR prior to checkerboard hybridization assay (10); and new incubation with fresh medium after CMP to enrich the bacterial sample (35).

This clinical study compared the sensitivity of methods for detection of bacteria, before and after CMP, by culture and PCR with previous WGA methods. To the best of our knowledge, this is the first clinical study that compares these assays to detect bacteria from root canals before and after endodontic treatment.

Several studies have demonstrated that culture-independent methods are more sensitive than culture-dependent methods in detecting bacteria (27, 38–41). This benefit can be justified due to some microorganisms' inability to grow under routine conditions, to microbial loads that are too low to detect, to environmental conditions within the treated root canal, and to the identification method of choice (38, 40).

In 2012, Paiva and coworkers compared the presence of bacteria in 27 samples of necrotic root canals of teeth with apical periodontitis, after CMP, by culture and PCR, detecting bacteria in 10 (37%) and 18 (66.7%) samples, respectively. Another study involving 50 samples from root-filled teeth also showed a great difference when comparing both detection methods, finding *E. faecalis*, in 40 (80%) by PCR and in eight (16%) by culture (41). So, according to these authors, the PCR method increased the sensitivity of bacterial detection in root canal samples ranging from 80% to 500%. In the present study, the detection by molecular method increased the sensitivity by 700% compared to the culture method, which can be attributed to PCR having been performed after DNA sample enrichment by WGA using isothermal strand displacement with Phi29 DNA polymerase.

Other microbiological studies demonstrated that WGA improved the sensitivity of detection of single copy genes, amplified products with less bias and also produced higher yields of amplified DNA when compared with Nested PCR, primer extension pre-amplification or degenerate oligonucleotide primed PCR (42, 43).

A limitation of this study, inherent to the PCR technique itself, is the persistence and detectability of DNA from dead bacterial cells for a variable amount of time, ranging from days to years (44, 45). Although we acknowledge this limitation, it is also known that a 60 s treatment with NaOCl is able to eliminate PCR amplification of *E. faecalis* DNA (45).

In conclusion, WGA increases the amount of bacterial DNA and enables detection of bacteria by PCR not otherwise identifiable by this method. The combination of these techniques represents an important tool to improve the detection of microorganisms from root canal samples after endodontic treatment using NaOCl as a CMP irrigant. This combination of techniques more accurately differentiates samples with very low bacterial loads from those with no bacteria, which can impact the choice of instruments, substances, and techniques used during endodontic treatment, consequently increasing the chances of a successful outcome.

## Data Availability

The original contributions presented in the study are included in the article, further inquiries can be directed to the corresponding author.
